# Supplementary material for: Probe-Based Real-Time qPCR Assays for a Reliable Differentiation of Capripox Virus Species
Source: Microorganisms. 2021 Apr 6;9(4):765. doi: 10.3390/microorganisms9040765 (PMC8067474; doi:10.3390/microorganisms9040765)
Supplement: Supplementary file 1 [file microorganisms-09-00765-s001.pdf]

Supplemental Table S1. Analytical sensitivity of the newly developed duplex real-time qPCR assays.

duplex assay	virus isolate	genome equivalents per µl	Cq-values species-specific assays															Cq-values pan Capripox
LSD-field-ORF126-Mix11-Taq-FAM <u>and</u> LSDvac-Mix5-HEX	LSDV-"Neethling" vaccine	10^6	15.8	15.9	15.8	15.9	16.0	16.2	16.1	15.9	15.8	16.0	15.6	15.8	15.7	15.9	15.8	16.4
		10^5	19.1	18.9	18.8	19.3	19.3	19.5	19.2	19.1	19.1	19.2	19.1	19.2	19.1	19.0	19.2	19.8
		10^4	22.2	22.1	22.1	22.7	22.5	22.7	22.6	22.4	22.3	22.3	22.3	22.4	22.3	22.3	22.4	23.1
		10^3	25.6	25.4	25.5	26.1	26.1	26.3	26.1	25.7	25.6	25.7	25.8	26.1	26.0	25.8	25.6	26.5
		10^2	28.9	28.9	29.0	29.3	29.2	28.6	29.7	29.1	29.2	29.3	29.0	29.4	29.3	29.2	29.3	29.9
		10^1	32.1	32.3	32.6	32.7	32.6	32.9	32.4	33.3	32.2	32.7	32.6	33.1	33.2	32.6	33.0	33.5
		10^0	36.1	35.9	37.1	no Cq	37.3	no Cq	no Cq	34.2	36.0	36.8	no Cq	35.3	37.1	37.6	36.0	37.0
	LSDV-"Macedonia 2016"	10^5	19.0	18.9	19.0	19.1	19.2	19.2	18.6	18.6	19.1	18.9	19.1	18.5	18.4	18.7	18.7	18.3
		10^4	21.9	22.0	22.1	21.9	22.3	22.5	21.9	21.9	21.9	22.0	22.1	22.2	22.1	21.8	21.8	21.7
		10^3	25.5	25.2	25.4	25.8	25.1	25.4	25.3	25.1	25.1	25.9	25.5	25.4	25.1	26.0	25.0	24.9
		10^2	28.6	28.9	28.8	28.8	28.6	28.9	28.2	28.2	28.4	28.8	28.6	28.9	28.8	29.0	28.4	28.3
		10^1	32.0	32.1	32.0	32.5	31.9	32.2	31.8	31.7	32.0	32.1	32.2	31.9	32.1	32.2	32.2	31.5
		10^0	35.5	36.6	36.0	34.9	35.8	34.7	37.7	36.1	36.1	35.5	35.0	35.0	36.7	35.4	34.0	35.0
SPPV-ORF041-Mix1-MGB-FAM <u>and</u> GTPV-ORF095-Mix1-MGB-HEX	SPPV-"V/104"	10^5	20.4	20.4	20.4	19.7	19.7	20.0	19.7	19.9	20.0	19.6	19.7	19.8	19.8	19.8	20.0	19.1
		10^4	23.7	23.6	23.7	24.0	23.3	23.1	23.6	23.3	23.2	23.5	23.2	23.1	23.4	23.3	23.3	22.5
		10^3	27.1	27.1	27.0	26.5	26.6	26.6	26.5	26.4	26.7	26.4	26.5	26.7	26.5	26.5	26.6	26.0
		10^2	30.6	30.6	30.4	30.3	29.8	29.8	30.1	29.7	29.8	30.2	29.7	29.8	30.0	30.0	29.9	29.0
		10^1	34.2	34.2	34.2	32.9	32.9	33.1	33.2	33.5	33.2	33.0	33.4	33.3	33.8	33.3	33.3	32.6
		10^0	38.0	38.4	38.7	no Cq	35.8	36.3	no Cq	36.7	36.5	37.3	36.5	39.2	36.1	36.6	36.4	38.6
	GTPV-"V/103"	10^4	24.0	23.5	23.5	22.5	22.5	23.4	22.6	22.5	23.4	22.7	22.6	23.3	22.7	22.6	23.7	21.2
		10^3	27.1	27.2	27.2	26.0	26.2	27.1	26.2	26.0	27.0	26.2	26.1	27.4	26.2	26.2	27.1	24.4
		10^2	31.0	31.0	31.0	29.5	29.4	30.1	29.5	29.4	29.9	29.6	30.1	27.8	29.5	29.6	29.7	27.8
		10^1	34.5	34.3	34.4	33.2	32.7	32.9	32.8	33.2	33.2	33.0	33.0	33.1	33.6	32.9	32.7	31.2
		10^0	38.0	38.8	38.1	37.4	36.9	36.8	36.1	35.8	35.9	36.6	36.6	36.4	37.0	36.3	36.3	34.4

For the newly developed assays, 15 technical replicates were performed. For comparison, the established pan Capripox qPCR (Bowden et al., 2008; Dietze et al., 2018) was used and the mean value of technical duplicates is presented.

Supplemental Table S2. Sensitivity of the duplex real-time qPCR assays consisting of LSD-field-ORF126-Mix15-LNA-FAM and LSDvac-Mix5-HEX. Pan capripox real-time qPCR of Bowden et al. (2008) with a modified probe published by Dietze et al. (2018) (Capri-p32-Mix1-Taq-FAM) with standard thermal cycling conditions served as reference method..

Genome equivalents/µl	number of positive replicates/overall replicates		
	LSD-field-ORF126-Mix15-LNA-FAM	LSDvac-Mix5-HEX	Capri-p32-Mix1-Taq-FAM
10^4	3/3	6/6	7/7
10^3	3/3	6/6	7/7
10^2	3/3	6/6	7/7
10^1	3/3	6/6	7/7
10^0	3/3	6/6	8/8

Supplemental Table S3. Specificity of the newly developed duplex assays for differentiation of LSDV field strains and LSDV vaccine strains consisting of LSD-field-ORF126-Mix15-LNA-FAM and LSDvac-Mix5-HEX. All samples were tested in duplicates. Capri-p32-Mix1-Taq-FAM (Capri-p32) served as reference assay. SPPV-ORF041-Mix1-MGB-FAM (SPPV) and GTPV-ORF095-Mix1-MGB-HEX (GTPV) were tested as duplex assays. Moreover, LSD-field-ORF126-Mix15-LNA-FAM (LSDfield-LNA) was tested as duplex assay in combination with LSDvac-Mix5-HEX (LSDvac). Cq-vaues are presented. Cut-off was set at Cq 40.0.

	sample	Capri-p32	SPPV	GTPV	LSDfield- LNA	LSDvac
SPPV samples	V/104	14.8	15.7	no Cq	no Cq	no Cq
	V/123	17.6	18.4	no Cq	no Cq	no Cq
	V/293	18.3	19.2	no Cq	no Cq	no Cq
	BH 50/19-01	27.1	27.9	no Cq	no Cq	no Cq
	BH 24/20-18	25.6	26.2	no Cq	no Cq	no Cq
	S-02 EDTA blood 14 dpi	27.0	28.3	no Cq	no Cq	no Cq
	S-09 EDTA blood 12 dpi	28.9	30.0	no Cq	no Cq	no Cq
	S-13 EDTA blood 10 dpi	26.9	28.1	no Cq	no Cq	no Cq
	S-02 serum 14 dpi	30.4	33.0	no Cq	no Cq	no Cq
	S-13 serum 10 dpi	33.1	34.6	no Cq	no Cq	no Cq
	S-05 nasal swab 12 dpi	14.1	15.1	no Cq	no Cq	no Cq
	S-12 nasal swab 14 dpi	18.9	19.6	no Cq	no Cq	no Cq
	S-06 oral swab 12 dpi	24.1	25.1	no Cq	no Cq	no Cq
	S-12 oral swab 10 dpi	27.3	28.3	no Cq	no Cq	no Cq
	S-11 lung	23.1	23.8	no Cq	no Cq	no Cq
	S-15 skin lesion prepuce	16.5	17.6	no Cq	no Cq	no Cq
	S-04 nasal septum	20.4	21.5	no Cq	no Cq	no Cq
	S-03 crust	13.9	14.8	no Cq	no Cq	no Cq
GTPV samples	V/103	16.2	no Cq	16.8	no Cq	no Cq
	V/279	23.9	no Cq	24.4	no Cq	no Cq
	BH 24/20-11	31.7	no Cq	33.4	no Cq	no Cq
	BH 24/20-12	34.9	no Cq	36.6	no Cq	no Cq
	Z/254 EDTA blood 7 dpi	26.1	no Cq	27.4	no Cq	no Cq
	Z/254 EDTA blood 10 dpi	24.7	no Cq	25.9	no Cq	no Cq
	Z/256 EDTA blood 13 dpi	28.1	no Cq	29.0	no Cq	no Cq
	Z/254 serum 10 dpi	34.7	no Cq	37.7	no Cq	no Cq
	Z/256 serum 13 dpi	36.3	no Cq	36.9	no Cq	no Cq
	Z/259 serum 23 dpi	30.6	no Cq	32.4	no Cq	no Cq
	Z/253 nasal swab 10 dpi	25.7	no Cq	27.0	no Cq	no Cq

	Z/256 nasal swab 10 dpi	18.5	no Cq	19.3	no Cq	no Cq
	Z/257 nasal swab 13 dpi	18.0	no Cq	18.6	no Cq	no Cq
	Z/259 nasal swab 21 dpi	23.3	no Cq	24.0	no Cq	no Cq
	Z/256 oral swab 15 dpi	24.4	no Cq	25.1	no Cq	no Cq
	Z/257 oral swab 13 dpi	28.3	no Cq	29.4	no Cq	no Cq
	Z/259 cervical lymph node	20.2	no Cq	22.4	no Cq	no Cq
	Z/255 lung	23.8	no Cq	25.3	no Cq	no Cq
	Z/260 skin chest	18.8	no Cq	19.4	no Cq	no Cq
	Z/254 skin nose	20.7	no Cq	22.0	no Cq	no Cq
	Z/253 trachea	19.8	no Cq	20.4	no Cq	no Cq
LSDV field isolate samples	V/96	24.7	no Cq	no Cq	24.0	no Cq
	V/101	14.7	no Cq	no Cq	14.2	no Cq
	V/107	15.3	no Cq	no Cq	15.2	no Cq
	V/281	13.9	no Cq	no Cq	13.5	no Cq
	BH 50/19-07	35.8	no Cq	no Cq	35.7	no Cq
	BH 24/20-13	28.0	no Cq	no Cq	27.5	no Cq
	BH 24/20-17	33.6	no Cq	no Cq	33.1	no Cq
	R/921 EDTA blood 10 dpi	28.3	no Cq	no Cq	28.2	no Cq
	R/276 EDTA blood 10 dpi	23.9	no Cq	no Cq	23.4	no Cq
	R/988 EDTA blood 10 dpi	27.6	no Cq	no Cq	27.3	no Cq
	R/988 serum 11 dpi	27.9	no Cq	no Cq	27.2	no Cq
	R/792 serum 9 dpi	31.9	no Cq	no Cq	31.2	no Cq
	R/280 nasal swab 13 dpi	30.0	no Cq	no Cq	29.3	no Cq
	R/981 nasal swab 11 dpi	23.6	no Cq	no Cq	23.1	no Cq
	R/988 nasal swab 11 dpi	22.9	no Cq	no Cq	22.4	no Cq
LSDV vaccine isolate samples	V/100	14.4	no Cq	no Cq	no Cq	13.7
	V/102	18.3	no Cq	no Cq	no Cq	17.8
	V/106	14.1	no Cq	no Cq	no Cq	13.3
	V/122	16.5	no Cq	no Cq	no Cq	15.9
	V/126	15.5	no Cq	no Cq	no Cq	14.9
	BH 50/19-03	29.9	no Cq	no Cq	no Cq	28.8
	BH 50/19-06	27.4	no Cq	no Cq	no Cq	26.7
	BH 24/20-15	30.4	no Cq	no Cq	no Cq	29.7
	R/129 nasal swab 7 dpi	33.1	no Cq	no Cq	no Cq	32.4

Supplemental Table S4. Appropriateness of duplex assay consisting of LSD-field-ORF126-Mix15-LNA-FAM and LSDvac-Mix5-HEX in cattle herds previously vaccinated against LSDV.

sample	Capri-p32	LSD-field-ORF126-Mix15-LNA-FAM		LSDvac-Mix5-HEX	
		single	duplex	single	duplex
V101 + V100	22,5	22,4	22,3	22,2	22,1
V101 1:100 + V100	23,6	29,1	29,5	22,1	22,1
V101 + V100 1:100	23,4	22,3	22,3	28,9	27,7
V107 + V106	23,3	23,2	23,2	23,0	22,9
V107 1:100 + V106	24,4	29,9	30,3	23,1	23,1
V107 + V106 1:100	24,2	23,2	23,2	30,2	28,8
V281 + V122	21,1	21,8	22,1	20,6	20,8
V281 1:100 + V122	21,8	28,5	29,6	20,6	20,9
V281 + V122 1:100	22,3	21,9	21,9	27,7	26,8
V96 + V102	24,8	24,9	25,0	25,2	25,2
V96 1:100 + V102	26,3	31,5	31,8	25,2	25,3
V96 + V102 1:100	25,3	24,7	24,9	32,0	31,4
